# TRIP13通过同源重组通路提高肺腺癌细胞的放射抗性

**DOI:** 10.3779/j.issn.1009-3419.2023.106.27

**Published:** 2024-01-20

**Authors:** Shutong GE, Runchuan GU, Xiongtao YANG, Changdan XU, Shijie WANG, Guangying ZHU

**Affiliations:** ^1^100029 北京，北京大学中日友好临床医学院放射肿瘤科（葛舒童，许长丹，朱广迎）; ^1^Department of Radiation Oncology, Peking University China-Japan Friendship School of Clinical Medicine, Beijing 100029, China; ^2^100029 北京，中日友好临床研究所（谷润川）; ^2^China-Japan Friendship Institute of Clinical Medicine, Beijing 100029, China; ^3^102202 北京，北京市昌平区医院肿瘤科（杨雄涛）; ^3^Department of Oncology, Changping District Hospital, Beijing 102202, China; ^4^100029 北京，中国医学科学院北京协和医学院中日友好医院放射肿瘤科（王诗杰，朱广迎）; ^4^Department of Radiation Oncology, China-Japan Friendship Hospital, Chinese Academy of Medical Sciences & Peking Union Medical College, Beijing 100029, China

**Keywords:** 肺肿瘤, 放射抵抗, TRIP13蛋白, 同源重组, Lung neoplasms, Radioresistance, TRIP13 protein, Homologous recombination

## Abstract

**背景与目的** 放疗是非小细胞肺癌（non-small cell lung cancer, NSCLC）最常用的治疗手段之一。然而，一部分肿瘤细胞对放射线的不敏感是放疗疗效差、患者预后不良的重要原因之一，探究放射抵抗背后的深层机制是解决这一临床难题的关键。本研究旨在寻找与肺腺癌（lung adenocarcinoma, LUAD）放射抵抗相关的分子，初步经数据库筛选锁定甲状腺素受体结合因子13（thyroid hormone receptor interactor 13, TRIP13）为主要研究对象，并探索TRIP13是否与LUAD的放射抵抗有关及具体机制，以期为临床接受放疗的LUAD患者的联合治疗提供理论依据和潜在靶点。**方法** 选取基因表达综合数据库（Gene Expression Omnibus, GEO）中的GSE18842、GSE19188和GSE33532共3个数据集，借助R 4.1.3软件分别筛选3个数据集中差异表达的基因（|log FC|>1.5, P<0.05），之后使用Venn diagram找出在3个数据集中共有的差异表达基因。随后，借助STRING在线工具和Cytoscape软件，对筛选出来的差异基因进行蛋白质相互作用分析和模块分析，借助Kaplan-Meier Plotter数据库对各基因进行生存预后分析，并确定TRIP13基因作为后续主要研究分子。随后，采用亚致死性剂量照射法对人LUAD细胞系H292进行多次X射线照射，以构建具有放射抗性的细胞系H292DR。采用细胞计数试剂盒-8（cell counting kit-8, CCK-8）实验和克隆形成实验验证H292DR细胞的放射抗性能力。Western blot检测H292细胞和H292DR细胞中TRIP13蛋白的表达水平。使用小干扰RNA（small interfering RNA, siRNA）沉默H292DR细胞中TRIP13蛋白的表达并进行Western blot检测。观察TRIP13沉默后H292DR细胞的克隆形成能力和迁移能力，随后检测共济失调-毛细血管扩张突变（ataxia telangiectasia mutated, ATM）蛋白等与同源重组密切相关的蛋白的表达水平变化。 **结果** 经多个GEO数据集筛选、外部数据集的验证以及生存分析发现，TRIP13在LUAD中高表达，并与接受过放疗的LUAD患者的不良预后有关；并且，TRIP13基因富集分析（gene set enrichment analysis, GSEA）的结果提示，TRIP13可能通过促进放疗后的同源重组修复而与LUAD放射抵抗有密切关联。经实验检测发现，TRIP13的表达在H292DR中上调，而沉默TRIP13后能够增加H292DR细胞对放射线的敏感性。 **结论** TRIP13与接受放疗后的LUAD患者的预后不良有关，可能是通过促进同源重组修复途径来介导LUAD细胞对放射线的抵抗。

## Abstract (en)

**Background and objective** Radiation therapy is one of the most common treatments for non-small cell lung cancer (NSCLC). However, the insensitivity of some tumor cells to radiation is one of the major reasons for the poor efficacy of radiotherapy and the poor prognosis of patients, and exploring the underlying mechanisms behind radioresistance is the key to solving this clinical challenge. This study aimed to identify the molecules associated with radioresistance in lung adenocarcinoma (LUAD), identified thyroid hormone receptor interactor 13 (TRIP13) as the main target initially, and explored whether TRIP13 is related to radioresistance in LUAD and the specific mechanism, with the aim of providing theoretical basis and potential targets for the combination therapy of LUAD patients receiving radiotherapy in the clinic. **Methods** Three datasets, GSE18842, GSE19188 and GSE33532, were selected from the Gene Expression Omnibus (GEO) database and screened for differentially expressed genes (|log FC|>1.5, P<0.05) in each of the three datasets using the R 4.1.3 software, and then Venn diagram was used to find out the differentially expressed genes common to the three datasets. The screened differential genes were then subjected to protein-protein interaction (PPI) analysis and module analysis with the help of STRING online tool and Cytoscape software, and survival prognosis analysis was performed for each gene with the help of Kaplan-Meier Plotter database, and the TRIP13 gene was identified as the main molecule for subsequent studies. Subsequently, the human LUAD cell line H292 was irradiated with multiple X-rays using a sub-lethal dose irradiation method to construct a radioresistant cell line, H292DR. The radioresistance of H292DR cells was verified using cell counting kit-8 (CCK-8) assay and clone formation assay. The expression levels of TRIP13 in H292 and H292DR cells were measured by Western blot. Small interfering RNA (siRNA) was used to silence the expression of TRIP13 in H292DR cells and Western blot assay was performed. The clone formation ability and migration ability of H292DR cells were observed after TRIP13 silencing, followed by the detection of changes in the expression levels of proteins closely related to homologous recombination, such as ataxia telangiectasia mutated (ATM) protein. **Results** Screening of multiple GEO datasets, validation of external datasets and survival analysis revealed that TRIP13 was highly expressed in LUAD and was associated with poor prognosis in LUAD patients who had received radiation therapy. And the results of gene set enrichment analysis (GSEA) of TRIP13 suggested that TRIP13 might be closely associated with LUAD radioresistance by promoting homologous recombination repair after radiation therapy. Experimentally, TRIP13 expression was found to be upregulated in H292DR, and silencing of TRIP13 was able to increase the sensitivity of H292DR cells to radiation. **Conclusion** TRIP13 is associated with poor prognosis in LUAD patients treated with radiation, possibly by promoting a homologous recombination repair pathway to mediate resistance of LUAD cells to radiation.

恶性肿瘤是危害公共健康、造成机体伤亡的主要因素之一。据2020年全球癌症数据^[1]^显示，肺癌的发病率和死亡率分别位于第二位和第一位，占每年总死亡人数的18%。在肺癌所有病理分型中，肺腺癌（lung adenocarcinoma, LUAD）最为常见，约占肺癌总确诊病例的50%^[2]^。手术、放疗、化疗以及免疫治疗等是目前临床治疗肺癌的常用策略。近些年来，随着立体定向放疗（stereotactic body radiotherapy, SBRT）等放疗技术的出现，极大程度提升了放疗的精准度并进一步扩大了放疗在临床上的应用^[3]^。目前，放疗在肺癌早期已达到了能和手术相媲美的治疗效果，为早期不可手术的肺癌患者带来了福音；同时，放疗也是肺癌各个阶段治愈性或姑息性治疗的重要策略之一^[4⇓-6]^。然而，目前临床上仅有一部分患者对放疗敏感，并且许多患者在治疗期间也出现了对放疗耐受、治疗后短时间内复发等问题，降低了放疗的疗效^[7]^。因此，进一步地探索肺癌放疗抵抗发生发展的潜在机制有助于改善肺癌放疗的治疗效果。

放射抗性的出现，是肿瘤细胞中多种因素和生理病理机制共同作用的结果。其中，肿瘤细胞在接受放射线刺激后，较强的DNA损伤修复能力是肿瘤放射抗性发生的重要机制之一^[8]^。真核生物的DNA损伤修复通路多种多样，非同源末端链接（nonhomologous end-joining, NHEJ）和同源重组（homologous recombination, HR）是DNA双链断裂（DNA double-strand break, DSB）后最为重要的两种修复方式^[9⇓⇓-12]^。肿瘤细胞内任意一种修复能力的增强均能减弱肿瘤对放射线的敏感性，寻找与肿瘤放疗抵抗相关的因素并施加干预，则有望解决临床放射线耐受的难题。本研究首先通过数据库分析发现了TRIP13与LUAD的放射抗性之间可能存在密切关系，且查阅目前已发表的文献^[13,14]^提示TRIP13与多种癌种的放射抗性以及DNA损伤后修复存在相关性。但TRIP13与LUAD的放射抗性之间的相关性尚不明确，目前未见报道。因此本研究旨在通过亚致死剂量照射法构建具有放射抗性的人LUAD H292DR细胞系，并依托此细胞系探究TRIP13与LUAD放射抵抗之间的相关性，以期为临床LUAD放疗耐受提供理论基础。

## 1 资料与方法

### 1.1 数据来源及数据分析

使用R 4.1.3软件从基因表达综合数据库（Gene Expression Omnibus, GEO）下载4个非小细胞肺癌（non-small cell lung cancer, NSCLC）的基因表达谱：GSE18842、GSE19188、GSE33532和GSE68465，共计809例样本，其中NSCLC样本660例，正常组织样本149例。将GSE18842、GSE19188、GSE33532 3个数据集用于差异基因的筛选与分析，仅包含LUAD病理亚型和正常组织样本信息的GSE68465数据集用于辅助验证。使用R 4.1.2软件进行基因名称转换后，利用Limma包筛选前3个数据集的差异表达基因，筛选标准为|log FC|>1.5，P<0.05。而后，使用Venn diagram在线工具（bioinformatics.psb.ugent.be/webtools/Venn/）获取3个数据集共同差异表达的基因。使用STRING在线网站（version-12-0.string-db.org/）分析基因之间的潜在关系，绘制蛋白质相互作用网络图。随后，通过使用Cytoscape软件中的MCONE聚类算法功能获得蛋白质相互作用网络中更为核心的模块。使用Kaplan-Meier Plotter数据库（kmplot.com/analysis/）分析核心模块中所涉及的相关基因与肺癌患者生存预后之间的关系。使用R 4.1.3软件的Survival包在GSE68465数据集中验证此前的分析结果，并使用enrichplot包进行基因富集分析。

### 1.2 细胞培养和相关试剂

人LUAD细胞系H292购于国家实验细胞资源共享平台——北京协和细胞资源中心。H292细胞培养于含有10%胎牛血清和1%双抗的RPMI-1640培养基中。RPMI-1640培养基（货号：SH30809.01）和胎牛血清（货号：SH30406.05）购于美国HyClone公司；青链霉素混合液（货号：15140122）和胰酶（货号：25200056）购于美国Gibco公司；转染试剂Lipofectamine 3000（货号：L3000015）购于美国Thermo Fisher公司；Opti-MEM I减血清培养基（货号：31985-070）购于美国Invitrogen公司。细胞计数试剂盒-8（cell counting kit-8, CCK-8）（货号：BS350B）购于Biosharp公司；Transwell小室（货号：353097）购于美国BD公司（Becton, Dickinson and Company）。GAPDH（货号：sc-47724，1:1000）、TRIP13（货号：sc-514285，1:1000）、p-ATM（货号：sc-47739，1:1000）、ATM（货号：sc-377293，1:1000）和REV7（货号：sc-135977，1:1000）抗体购于Santa cruz公司；RAD51（货号：A00088，1:1000）购于武汉Boster生物公司，HRP标记的山羊抗鼠（货号：ab6789，1:10,000）、兔IgG二抗（货号:ab6721，1:10,000）购于美国Abcam公司。

### 1.3 人LUAD H292细胞系放射抗性表型构建方法

取生长状态良好的H292细胞，使用160 kV X线生物辐照仪（RAD SOURCE 2000pro）分别对细胞给予0、2、4、6、8和10 Gy不同的放射线剂量照射。将照射后的细胞重新放于细胞培养箱中培养，此后每隔2-3 d进行传代培养并确定H292细胞的亚致死性剂量为6 Gy。每照射一次细胞后需待细胞逐渐恢复至正常的生长速度后再给予下一次放射线照射，亚致死性照射剂量重复照射6次以上，大约3个月完成照射后停止照射并稳定传代5代以上即视为具有放射抗性表型，并将其称之为H292DR细胞系。

### 1.4 CCK-8细胞增殖实验验证放射抗性表型

取对数生长期的H292细胞和H292DR细胞，以每孔2000个/100 μL的细胞密度接种于96孔板中，测量0 h时间点的450 nm波长处的吸光度值并记录。翌日，待细胞贴壁24 h后，对孔板给予单次6 Gy的X射线照射。此后，依次测量24、48、72和96 h时间点的450 nm波长处吸光度值，绘制折线图并进行计算。

### 1.5 克隆形成实验验证放射抗性表型

取对数生长期的H292细胞和H292DR细胞，按计划照射的0、2、4、6、8 Gy放射线剂量分别铺300、600、900、1200和1500个细胞于12孔板中，每个剂量每组设置3个复孔。翌日，不同组分别接受不同剂量的放射线照射。此后，放回培养箱中静置培养8-10 d直至形成肉眼可见的克隆。结晶紫室温染色15 min后洗净并拍照计数。

### 1.6 Western blot检测蛋白表达

取对数生长期的H292细胞和H292DR细胞，使用RIPA裂解液提总蛋白并进行BCA蛋白浓度定量，加入5×Loading buffer煮沸使蛋白变性，而后采用SDS-聚丙烯酰胺凝胶电泳以分离目标蛋白，100 V恒压转膜，5%脱脂牛奶室温封闭1 h后，一抗4 ^o^C孵育过夜，翌日TBST洗3次后室温孵育二抗1 h，而后曝光显影。

### 1.7 小干扰RNA（small interfering RNA, siRNA）转染细胞

转染前一天，将H292DR细胞按2×10^5^个/mL铺于12孔板中，次日细胞密度约为70%。将细胞分为阴性对照组（转染siRNA-negative control，即NC）、si1组（转染靶向TRIP13的第1条siRNA）和si2（转染靶向TRIP13的第2条siRNA）3组。按照Lipofectamine 3000说明书将siRNA转染至H292DR细胞中。转染6-8 h后更换成完全培养基。此后收取24、48、72 h时间点的细胞，进行Western blot验证转染效果。靶向TRIP13的siRNA及对照siRNA的序列由上海吉凯基因化学技术有限公司合成。TRIP13的2条有效干扰靶点序列（5’-3’）分别为AGCTACTCAACAGACATAATATT和CTCGATTATGTGATGACAACTTT。

### 1.8 Transwell细胞迁移实验

取24孔板，孔板内加入完全培养基，将Transwell小室轻柔放入。取siRNA处理后的H292DR细胞，使用无血清培养基清洗3次并重悬后种至小室中。随后将放有Transwell小室的孔板置于孵箱中。48 h后，取出小室，使用结晶紫染液进行室温染色20 min，清洗晾干后置于显微镜下拍照计数。

### 1.9 统计学方法

采用SPSS 26.0和GraphPad Prism 8.0进行本研究的数据分析及绘图。采用Kaplan-Meier方法分析TRIP13与患者的总生存期（overall survival, OS）的关系，采用Spearman相关分析检测TRIP13基因与其他基因之间的相关性。单因素和多因素Cox回归分析评估多个因素对LUAD患者OS的影响。两组间计量资料的比较采用t检验。P<0.05为差异具有统计学意义。

## 2 结果

### 2.1 筛选3个GEO数据集以获得共同差异表达基因

本次使用的所有GEO数据集的信息如表1所显示。使用Limma包分析GSE18842、GSE19188、GSE33532 3个数据集分别得到418、203和371个表达上调的基因，以及775、564和689个表达下调的基因。使用Venn diagram绘制Venn图后获得了521个共同差异表达的基因，其中有164个共同表达上调的基因，357个共同表达下调的基因（图1）。

**表 1:** 本研究所用的GEO数据集信息

GEO dataset	Cancer tissue	Normal tissue	Total
GSE18842	46	45	91
GSE19188	91	65	156
GSE33532	80	20	100
GSE68465	443	19	462
GSE37745	196	0	196

GEO: Gene Expression Omnibus; GSE: Gene Expression Omnibus Series.

**图 1:**
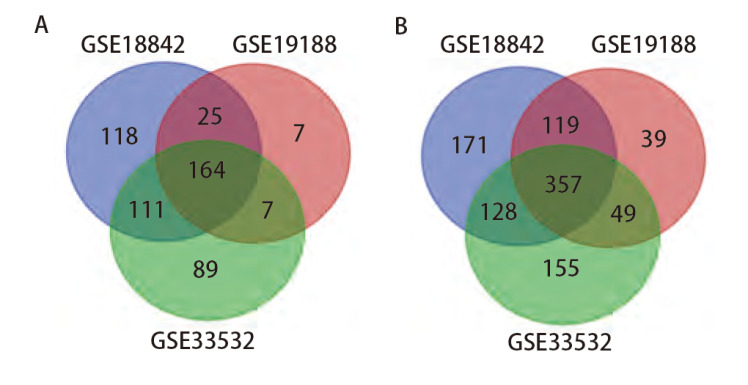
3个数据集共同表达上调的164个基因和共同表达下调的357个基因。 A：3个数据集共同表达的上调基因的数量；B：3个数据集共同表达的下调基因的数量。

### 2.2 差异基因的初步生存分析发现TRIP13与LUAD患者的不良预后有关

将上述获得的521个差异表达的基因上传至STRING在线网站，绘制蛋白质相互作用网络图，联合使用Cytoscape软件，进一步可视化基因间的相关性，并使用MCONE功能进一步获得了一个包含85个基因的核心模块（图2），这85个基本全部为表达上调的差异基因。随后，使用Kaplan-Meier Plotter网站对该85个基因进行生存分析，对某一基因进行数据分析过程中，均以该基因表达量的中位值为界，将患者分为高表达组和低表达组。结果显示，该85个基因中84个基因均与NSCLC患者的OS有关，其中75个基因与LUAD患者的OS有关（表2）。在该条件下进一步将条件限制在接受放疗的肺癌患者人群中，发现TRIP13的表达仍与接受放疗的肺癌患者的生存预后有关（图3A）。

**图 2:**
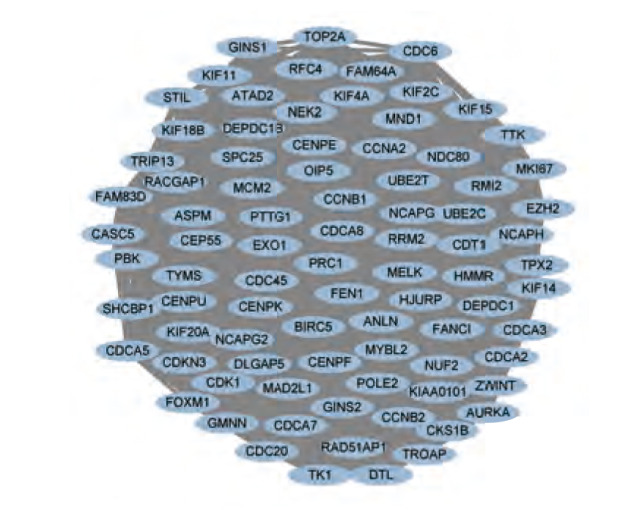
Cytospace分析后所得的85个核心基因

**表 2:** Kaplan-Meier Plotter分析84个基因与与LUAD生存预后之间的关系

Category	Genes	Count
With significant relationship between gene expression and survival (P<0.05)	KIF14, MKI67, CENPE, TYMS, KIF4A, PTTG1, MCM2, NCAPG, PRC1, CENPU, RMI2, DEPDC1B, TPX2, CDKN3, EZH2, TOP2A, DEPDC1, CDT1, RRM2, CENPF, FOXM1, CASC5, NDC80, PBK, DTL, KIF11, CCNB1, TK1, KIF2C, ZWINT, FAM64A, MELK, CDC45, DLGAP5, CCNA2, CDCA2, MND1, CDCA8, CDCA5, NCAPH, CCNB2, KIAA0101, GMNN, MAD2L1, NCAPG2, CDC20, KIF18B, SPC25, OIP5, UBE2C, FANCI, MYBL2, TTK, UBE2T, AURKA, NEK2, ATAD2, CDC6, KIF15, RFC4, TRIP13, ANLN, NUF2, GINS1, RACGAP1, BIRC5, KIF20A, SHCBP1, TROAP, CDCA3, CEP55, EXO1, GINS2, ASPM, HJURP	75
Without significant relationship between gene expression and survival (P>0.05)	CDCA7, RAD51AP1, FAM83D, CENPK, POLE2, STIL, FEN1, HMMR, CKS1B	9

LUAD: lung adenocarcinoma.

**图 3:**
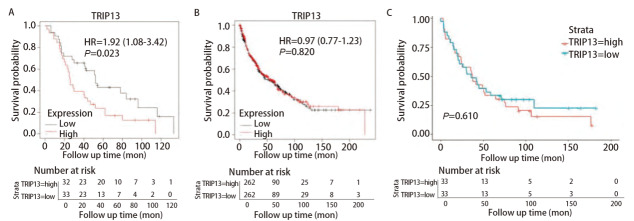
TRIP13的表达和肺癌患者生存预后之间的关系。 A：Kaplan-Meier Plotter数据库显示的TRIP13的表达与接受放疗的肺癌患者（n=65）的生存预后有关；B、C：Kaplan-Meier Plotter数据库（n=524）和GSE37745数据集（n=66）双重证明TRIP13与LUSC患者的生存预后无明显的关联。

而据Kaplan-Meier Plotter数据库分析显示，TRIP13表达量与肺鳞癌（lung squamous cell carcinoma, LUSC）患者的生存预后无关（P=0.820，图3B）；使用含有66例LUSC的GSE37745数据集再次进行验证，结果同样显示TRIP13的表达量与LUSC患者的生存预后无关（P=0.610，图3C）。这种在肺癌不同病理亚型中的差异可能是因为肿瘤异质性所导致的，故此后将研究重点放在了TRIP13与LUAD之间的关系上。

为验证TRIP13表达量与LUAD以及接受放疗的LUAD患者生存预后之间的关系，又选择在含有443例LUAD样本和19例正常组织样本表达数据的GSE68465数据集进行了生存分析。此外，由于R软件在进行生存分析过程中自动将TRIP13表达量的中位值舍弃，并将剩余数据分为TRIP13表达量高和表达量低的两组，故图中显示的总病例数比实际病例数少1例（图4）。结果显示，TRIP13表达水平与LUAD患者的OS率有关（P<0.001），TRIP13表达量越高的患者，OS率越低。为进一步观察TRIP13基因的表达是否与接受过放疗的LUAD患者的生存预后相关，筛选了该数据集中65例接受过放疗的LUAD患者，对这一部分人群再次进行生存分析，发现TRIP13的基因表达量与这65例患者的OS率有关（P=0.037）。这些结果表明，TRIP13或许可作为LUAD患者（包含既往接受过放疗）预后的分子标志物。

**图 4:**
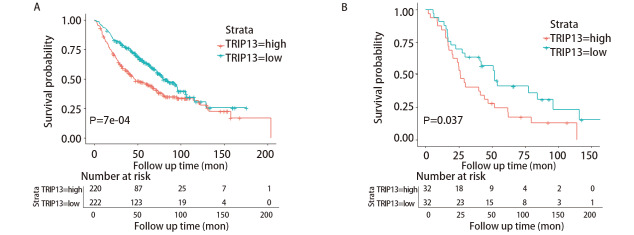
GSE68465数据集验证TRIP13与LUAD（n=442）（A）以及其中接受放疗的LUAD患者（n=64）（B）的生存预后之间的关系。因R软件在进行生存分析过程中，自动将TRIP13表达量的中位值舍弃，再将剩余数据分为TRIP13表达量高和表达量低的两组，故图中显示的总病例数比实际病例数少1例。

### 2.3 TRIP13可能参与DSB后的重组修复

为进一步观察TRIP13基因可能参与的信号通路，在仅含有正常肺组织和LUAD组织的mRNA数据的GSE68465数据集中，通过使用R软件的enrichplot包对TRIP13基因进行了基因富集分析，结果发现，TRIP13基因与细胞周期检查点信号通路、DNA复制以及DSB的HR修复有密切关系（图5）。其中，HR修复是放射线作用于癌细胞并引起癌细胞发生DSB事件后最主要的修复方式之一，也与肿瘤的放射抵抗有密切关系。由此可见，TRIP13可能与LUAD的发生发展有关，并且很大程度上可能与LUAD的放疗抗性有关，或可成为临床上LUAD治疗的潜在靶点和预后标志物。

**图 5:**
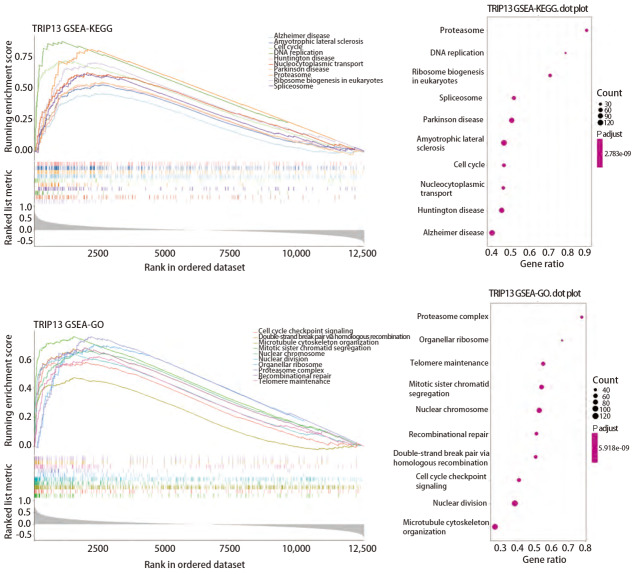
使用GSE68465数据集对TRIP13进行单基因的GSEA分析

根据以上分析提示的TRIP13与DNA损伤修复之间可能存在的联系，通过TIMER在线数据库分析了TRIP13基因与HR信号通路的多个基因之间的相关性，结果（Spearman相关分析）发现，在LUAD中TRIP13与HR通路的多个基因之间存在明显的正相关，例如重组酶RAD51（RAD51 recombinase, RAD51）基因（r=0.780, P<0.001）、乳腺癌1号（breast cancer 1, BRCA1）基因（r=0.782, P<0.001）、核酸外切酶I（exonuclease 1, EXO1）基因（r=0.803, P<0.001）以及乳腺癌2号（breast cancer 2, BRCA2）基因（r=0.591, P<0.001）等，提示在LUAD中，TRIP13可能会促进放疗后的HR，与肿瘤的放疗抵抗、患者的不良预后有关。

### 2.4 TRIP13表达量与接受放疗的LUAD患者临床预后有关

Kaplan-Meier生存分析的结果显示，TRIP13高表达组的患者预后较差，提示TRIP13可作为LUAD患者临床预后的潜在指标。GSE68465数据集中包含65例既往接受过辅助放疗的NSCLC患者的临床数据，对这些临床数据进行单因素和多因素Cox回归分析（表3），以更好地了解TRIP13与既往接受过放疗的LUAD患者的生存预后之间的关系，结果表明TRIP13表达量、肿瘤原发灶-淋巴结-转移（tumor-node-metastasis, TNM）分期、是否接受过辅助化疗和肿瘤组织分化程度对LUAD患者的OS有影响（P<0.05），其中TRIP13高表达、较晚的TNM分期（III期）和较差的肿瘤组织分化程度是影响患者预后的危险因素[风险比（hazard ratio, HR）>1，P<0.05]，患者的OS较短；而接受过辅助化疗显示是患者的保护性因素（HR=0.510, 95%CI: 0.280-0.932），患者的OS较长。然而，将单因素分析显示的与患者预后有关的因素纳入多因素Cox分析，仅TNM分期对患者的预后有影响（HR=2.112, P=0.014）；TRIP13的表达量虽不再对患者的OS有影响（P=0.068），但仍在一定程度上是LUAD患者的危险因素，TRIP13高表达患者的死亡风险是低表达患者的1.714倍。

**表 3:** GSE68465数据集中65例接受过放疗的LUAD患者预后因素的单因素和多因素Cox回归分析

Parameters		Univariate analysis			Multivariate analysis	
		HR (95%CI)	P		HR (95%CI)	P
Gender	Female	Reference				
	Male	1.126 (0.622-2.040)	0.695			
Age (yr)	<60	Reference				
	≥60	1.280 (0.696-2.353)	0.428			
TNM stage	l-ll	Reference				
	lll	2.170 (1.230-3.829)	0.008		2.112 (1.161-3.850)	0.014
Differentiation	Well-moderate	Reference				
	Poor	2.011 (1.127-3.589)	0.018		1.348 (0.737-2.465)	0.330
Adjuvant chemotherapy	No	Reference				
	Yes	0.510 (0.280-0.932)	0.029		0.531 (0.280-1.010)	0.054
Smoking history	No	Reference				
	Yes	2.235 (0.883-5.652)	0.090			
TRIP13 expression	Low	Reference				
	High	1.813 (1.026-3.206)	0.041		1.714 (0.960-3.061)	0.068

HR: hazard ratio; CI: confidence interval; TNM: tumor-node-metastasis.

### 2.5 构建具有放射抗性的人LUAD细胞系

为确定TRIP13分子与LUAD放疗抵抗之间的相关性，首先构建了具有放射抗性表型的人LUAD细胞系H292DR。此后为了验证放射抗性表型的成功构建，对H292DR细胞和H292细胞进行了CCK-8细胞增殖实验和克隆形成实验验证。相较于H292细胞而言，H292DR细胞形成的克隆更大更多；由于高剂量组在开始时所接种的细胞多，虽6和8 Gy等高剂量组相较于低剂量组而言形成的克隆数较多，但存活分数随着放射剂量的升高而逐渐降低；并且随着X射线剂量的增加，相较于H292细胞而言，H292DR的克隆形成率更高，两组具有统计学差异（图6A、6B）。CCK-8实验结果同样表明，H292DR在不同剂量的X射线照射下具有更强的细胞增殖能力（图6C），表明放射抗性细胞系构建成功。显微镜明场下观察，H292DR细胞在形态上也发生了些许改变，体积稍变大（图6D）。随后，为了观察在具有放射抗性的细胞中TRIP13蛋白的表达情况，Western blot检测H292细胞和H292DR细胞中TRIP13的蛋白含量，发现H292-IR细胞中TRIP13蛋白水平相较于H292细胞而言有轻微升高的趋势，但没有统计学差异（图6E）。

**图 6:**
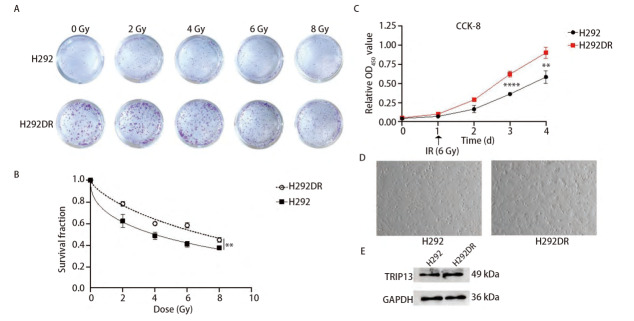
构建放射抗性的LUAD细胞系并加以验证，检测TRIP13蛋白的表达情况。 A、B：克隆形成实验验证H292DR的放射抗性表型，并绘制存活曲线；C：CCK-8细胞增殖实验验证H292DR的放射抗性表型；D: 显微镜明场视野下观察H292和H292DR细胞（×40）；E：Western blot检测H292和H292DR细胞中TRIP13蛋白的表达情况。**P<0.01, ****P<0.0001。

### 2.6 沉默TRIP13后能够减弱H292DR细胞的克隆形成和迁移能力

为了观察TRIP13在放射抵抗的LUAD细胞中的作用，使用2条siRNA沉默H292DR细胞中的TRIP13。Western blot检测siRNA作用24、48和72 h后TRIP13的表达情况，发现在3个时间点，相较于NC组而言，2条siRNA均能明显降低H292DR细胞中TRIP13蛋白水平的表达（图7A）。随后，再次重复对TRIP13分子的敲减，并收取siRNA作用24 h后的H292DR细胞进行克隆形成实验和Transwell细胞迁移实验。

**图 7:**
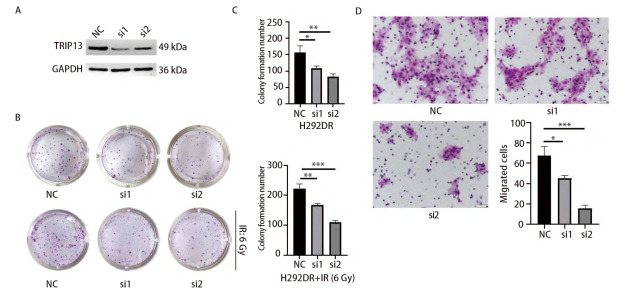
沉默TRIP13的表达后对LUAD细胞的克隆形成和迁移能力的影响。 A：Western blot检测siRNA沉默TRIP13蛋白表达的效率，图中展示的是siRNA作用24 h后收取蛋白进行的检测；B、C：siRNA处理后的H292DR细胞进行克隆形成实验，上面一行不给予放射线处理，下面一行给予单次6 Gy放射线处理，并对克隆形成结果进行统计学分析；D：siRNA处理H292DR细胞后进行Transwell细胞迁移实验，并对结果进行统计学分析。*P<0.05, **P<0.01，***P<0.001。

克隆形成实验分成两组，一组不接受放射线处理，每孔铺300个细胞至12孔板中；另一组，每孔铺500个细胞至12孔板中，次日在细胞贴壁24 h后给予单次6 Gy的放射线处理。此后，两组继续培养直至形成肉眼可见的克隆。实验结果表明，沉默TRIP13后，不论是否有放射线的干预，均能降低放射抗性H292DR细胞的克隆形成能力，对照组和siRNA敲减组之间差异具有统计学意义（P<0.05，图7B、7C）。细胞迁移实验结果显示，沉默H292DR细胞中TRIP13表达后，能够明显削弱H292DR细胞的迁移能力（P<0.05，图7D），这一结果与平板克隆的实验结果相吻合。以上结果表明，沉默TRIP13不仅可以增加具有放射抗性的H292DR细胞对放射线的敏感性，还可抑制其迁移能力。

### 2.7 沉默TRIP13后削弱放射抗性LUAD细胞的HR修复能力

由于此前对TRIP13在LUAD中的单基因富集分析的结果提示，TRIP13很有可能与HR修复有密切关系。为进一步地观察沉默TRIP13后具有放射抗性表型的LUAD细胞的DNA损伤修复能力是否会受到影响。于是，在使用siRNA成功敲低TRIP13后，同步检测ATM、p-ATM、RAD51以及REV7这4个HR修复通路关键分子的蛋白表达变化，结果（图8）显示，沉默TRIP13的表达后总ATM的表达没有明显变化，而p-ATM（S1981）的磷酸化水平、RAD51和REV7的表达水平均出现了明显的降低。该结果提示，TRIP13可能与HR修复密切相关，沉默TRIP13后或可通过削弱LUAD细胞的DNA修复能力来增加放射抗性细胞对放射线的敏感性。

**图 8:**
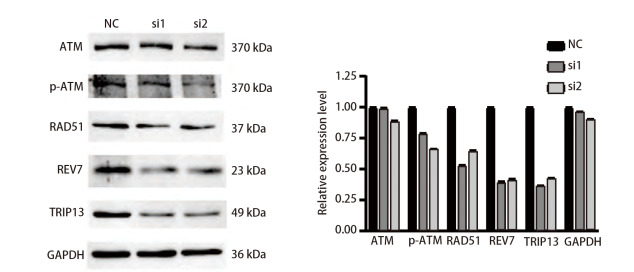
沉默TRIP13的表达对H292DR细胞中与同源重组修复相关蛋白表达的影响

## 3 讨论

肿瘤细胞是一个异质性很强的群体，部分肿瘤细胞对放射线不敏感，在反复放射线照射的刺激下获得生存优势，放射抗性的相关基因组突变得以积累，是导致临床上恶性肿瘤患者放疗疗效不佳、生存预后差的一个重要原因。近年来，国内外也已有大量的基础研究^[15,16]^围绕着恶性肿瘤的放疗抵抗问题开展。在本研究中，我们首先通过分析GEO数据库中与肺癌有关的数据，经筛选以及外部数据集验证，发现TRIP13基因在NSCLC中表达上调，并与患者的不良预后有关。进一步分析发现，TRIP13基因与NSCLC中的LUAD亚型关系更为密切；并且在接受过放疗的LUAD人群中，TRIP13基因的高表达患者的总生存率亦有显著的降低（P<0.05）。对TRIP13进行GSEA分析发现，TRIP13可能参与DSB后的HR修复，提示这可能是接受放疗后TRIP13高表达的LUAD人群预后不良的原因。

TRIP13是ATP酶中AAA(+) ATP酶家族成员之一，参与减数分裂和有丝分裂，在细胞分裂过程中发挥了重要作用^[17,18]^。在减数分裂的G_2_前期，大量DSB的积累引发了染色体重组检查点的激活，TRIP13参与调控同源染色体重组并促进了DSB修复，以保证细胞分裂的顺利进行；而在有丝分裂期间，TRIP13则主要参与了检查点信号通路、DSB修复等，此外，TRIP13还通过改变有丝分裂阻滞缺陷蛋白2（closed-mitotic arrest deficient protein 2, C-MAD2）构象，释放细胞分裂周期蛋白20（cell division cycle 20, CDC20），以激活有丝分裂后期促进复合物/细胞周期体（anaphase-promoting complex/cyclosome, APC/C）来推动细胞分裂进入后期^[17,19]^。然而，近些年来，越来越多的研究^[20⇓-22]^发现TRIP13也参与到癌症发生发展中。TRIP13的表达量与恶性肿瘤患者的不良预后以及与放疗、化疗敏感性之间的关系也已在文献^[13]^中得到了初步的报道与证实。多项研究^[13,14,20⇓-22]^表明TRIP13在不同的癌症类型（如乳腺癌、前列腺癌、肺癌、骨髓瘤、头颈癌等）中存在过表达或异常扩增，提示它可能具有致癌特性。例如，Kang等^[23]^通过在36例NSCLC患者的样本中进行高分辨率微阵列比较基因组杂交技术，以期发现早期NSCLC遗传学事件，结果显示50%的NSCLC患者存在TRIP13拷贝数异常，而此比例在I期NSCLC患者中则更高（68%）。Clairmont等^[14]^的研究表明，TRIP13通过催化前NEHJ因子——REV7发生失活构象改变，可抑制NHEJ并促进HR的发生；并发现TRIP13的高表达促进了HR、介导了聚（腺苷二磷酸）-核糖聚合酶[poly ADP (adenosine diphosphate)-ribose polymerase, PARP]抑制剂的耐药性，与BRCA1缺陷型乳腺癌患者的不良预后相关。Banerjee等^[13]^的研究则是发现TRIP13在头颈癌中过表达，促进癌症的发生发展，并通过促进NHEJ来介导癌细胞对顺铂以及放射线的耐受性，造成癌细胞对放化疗的抗性。而Wang的研究团队^[24]^则通过晶体结构鉴定，预测并验证了靶向TRIP13分子抑制剂——DCZ0415的存在，该抑制剂通过抑制NEHJ过程和核因子κB（nuclear factor kappa-B, NF-κB）活性，发挥了良好的抗骨髓瘤治疗活性。

目前已发表的研究^[20,25]^结果，也与我们通过生存分析所显示的TRIP13的高表达与LUAD患者的不良预后有关相吻合，额外的生存分析以及针对TRIP13的单基因富集分析结果提示TRIP13的异常扩增可能通过促进放疗后的DNA损伤修复，导致接受放疗的LUAD患者预后不良。但目前TRIP13与LUAD的放疗抗性之间的关系尚无报道。

为了明确TRIP13分子和LUAD放疗抗性之间的关系，我们首先通过亚致死剂量照射法构建了具有放射抗性的人LUAD细胞系，并将其称之为H292DR。通过检测H292细胞和H292DR细胞中TRIP13蛋白的表达，发现TRIP13蛋白的表达水平在H292DR有轻微的升高趋势。随后，通过设计siRNA靶向敲低H292DR细胞中TRIP13的表达，观察到，敲减TRIP13的表达后能显著降低H292DR细胞的克隆形成能力和迁移能力（P<0.05）。由于前期数据分析结果提示TRIP13可能参与促进DSB后的HR修复，而ATM、REV7、BRCA2和RAD51等是参与DSB后HR修复的重要分子。其中，ATM存在多个自磷酸化位点，如丝氨酸367位点（ser-367）、丝氨酸1893位点（ser-1893）、丝氨酸1981位点（ser-1981）、丝氨酸2996位点（ser-2996）和苏氨酸1885（thr-1885）位点，前4个位点在DSB作用下均会发生自磷酸化，而ser-1981位点的磷酸化是最为特异的，常被用作ATM激活的标志，反映DSB后HR修复信号通路的激活^[10,26]^。我们将H292DR细胞中的TRIP13分子敲低后，检测了与HR密切相关的分子的蛋白表达水平，观察到TRIP13敲减后细胞中的总ATM 水平没有发生变化，而p-ATM（ser-1981）、RAD51和REV7的表达水平均发生了降低，表明TRIP13的敲减可能使得具有放射抗性的人LUAD细胞的HR修复能力降低，进而提升LUAD细胞对放射线的敏感性。但本研究所进行的验证较少，仅围绕着敲减TRIP13后H292DR细胞的增殖能力和迁移能力减弱以及HR信号通路相关蛋白表达降低进行论述，而免疫荧光观察γH2AX和中性彗星实验等验证细胞DSB后修复能力的实验暂未进行。其中，γH2AX是细胞发生DSB后快速响应的蛋白之一，表现为在胞核内的聚集，免疫荧光观察γH2AX数量的多少可用于衡量DNA发生双链断裂的程度^[27]^，而中性彗星实验通过衡量彗星尾矩的长短亦可反映细胞的DSB程度。此外，本研究的数据来源仅局限于GEO公共数据库，未结合临床病例及临床组织样本进行分析，未实现由基础实验到临床的衔接，此部分有待进一步完善。本研究后续将继续增加实验验证以明确TRIP13和LUAD放疗抵抗之间的相关性。

综上，我们发现了TRIP13在具有放射抗性的人LUAD细胞中发挥重要作用，可能通过影响DSB后的HR修复进而导致LUAD细胞对放射线的不敏感，并与临床上LUAD患者的不良预后有关联。因此，对于接受放疗的LUAD患者而言，TRIP13表达水平较高可能与患者放疗疗效不佳、预后不良有关联。TRIP13或可作为临床上判断LUAD患者，尤其是接受放疗患者的预后的一个潜在生物标志物。
